# Editorial: Neglected and Under-Researched Parasitic Diseases of Veterinary and Zoonotic Interest

**DOI:** 10.3389/fvets.2021.701848

**Published:** 2021-06-11

**Authors:** Olivier Sparagano, Lise Roy, Annunziata Giangaspero

**Affiliations:** ^1^Department of Public Health and Infectious Diseases, City University of Hong Kong, Hong Kong, China; ^2^Department of Biology, Ecology and Environment, Université Paul Valéry, Montpellier III, Montpellier, France; ^3^Department of Agriculture, Food, Natural Resources and Engineering, University of Foggia, Foggia, Italy

**Keywords:** neglected parasitic disease, veterinary parasitic disease, zoonotic parasitic disease, parasites, zoonosis

We are delighted to have contributed as Guest Editors to this special issue entitled: “Neglected and under-researched parasitic diseases of veterinary and zoonotic interest.” This Special issue represents 17 manuscripts published from 94 authors (representing 17 countries) which, so far, encountered thousands views.

Neglected or poorly understood diseases can have a global impact as we have seen with COVID-19 reaching a global pandemic level very quickly. Interestingly, the parasitic diseases presented in this Special Issue are already having a human, animal, societal, and zootechnical impacts in their own rights but also sometimes due to the diseases that such parasites can carry, contributing to further morbidity and mortality.

In veterinary and human parasitology, many diseases have been excluded from international debate. Certain diseases are recognized as global emergencies, while others are considered niche or have dropped out of scientific interest. This imbalance may be due to two main reasons, which are independent from their health interest. First, the arbitration modalities of research funding and the evaluation of researchers (notably the mechanism of citation indices) in Western countries are such that they concentrate forces on a small number of parasites affecting the greatest number of people and tend to discourage studies on parasites that affect fewer people or people in countries located in other regions of the world, regardless of their health impact. Second, some parasites are elusive due to special lifestyles and thus largely unknown.

In this Special Issue you will learn about many neglected parasites (mites, protozoans, helminths) found in pets (cats and dogs), wildlife (chamoix), livestock animals (cattle, buffaloes, horses, poultry), and highlighting state-of-the-art techniques in potential drug targets or diagnostic markers. Some manuscripts are also highlighting the epidemiological aspects, and transmission risks for human populations as well.

This Research Topic discuss contributions on the parasitic diseases whose economic and health importance and spread is threatened due to the following reasons:

- Underestimated due to their particular lifestyle that makes them elusive and difficult to study (e.g., poultry ectoparasites, including Dermanyssus gallinae and Ornithonyssus bursa and their zoonotic role);
- Erroneously considered eradicated (e.g., habronematidosis in equids);
- Lesser studied due to prevalence in low-income countries (e.g., Cryptosporidium spp., Sarcocystis in horses, Dracunculus in dogs);
- Often ignored (e.g., eimeriosis and neosporosis in ruminants, cestodosis in dogs) and in unexplored hosts (e.g., Eimeria in wild animals, Neospora and Toxoplasma in ducks);
- Rare and unrecognized (e.g., feline angiostrongylosis);
- Lack of/incorrect treatment (e.g., babesiosis in humans).

These neglected diseases should be taken into serious consideration. As we have seen with Covid-19, unknown, poorly studied or emerging diseases can have a global impact on humans, animals, and the environment and the neglected parasitic diseases presented in this Special Issue deserve further monitoring and research development.

We hope you will enjoy reading and reflecting on these important topics and consider challenging such knowledge gap while developing new integrated and multidisciplinary collaborations.

## Author Contributions

All authors listed have made a substantial, direct and intellectual contribution to the work, and approved it for publication.

## Conflict of Interest

The authors declare that the research was conducted in the absence of any commercial or financial relationships that could be construed as a potential conflict of interest.

